# Novel Cage-Like Hexanuclear Nickel(II) Silsesquioxane. Synthesis, Structure, and Catalytic Activity in Oxidations with Peroxides

**DOI:** 10.3390/molecules21050665

**Published:** 2016-05-19

**Authors:** Alexey N. Bilyachenko, Alexey I. Yalymov, Lidia S. Shul’pina, Dalmo Mandelli, Alexander A. Korlyukov, Anna V. Vologzhanina, Marina A. Es’kova, Elena S. Shubina, Mikhail M. Levitsky, Georgiy B. Shul’pin

**Affiliations:** 1 Nesmeyanov Institute of Organoelement Compounds, Russian Academy of Sciences, Vavilov Str. 28, 119991 Moscow, Russia; alexyalymov@gmail.com (A.I.Y.); shulpina@ineos.ac.ru (L.S.S.); alex@xrlab.ineos.ac.ru (A.A.K.); vologzhanina@mail.ru (A.V.V.); marinaeskovskaya@gmail.com (M.A.E.); shu@ineos.ac.ru (E.S.S.); levitsk@ineos.ac.ru (M.M.L.); 2 Inorganic Chemistry Department, People’s Friendship University of Russia, Miklukho-Maklay Str. 6, 117198 Moscow, Russia; 3 Center of Natural and Human Sciences, Federal University of ABC (UFABC), Santa Adélia Street, 166, Bangu, Santo André, SP 09210-170, Brazil; dalmo.mandelli@ufabc.edu.br; 4 Chemistry Department, Russian National Research Medical University, Ostrovitianov str. 1, 117997 Moscow, Russia; 5 Semenov Institute of Chemical Physics, Russian Academy of Sciences, ul. Kosygina, dom 4, 119991 Moscow, Russia; 6 Chair of Chemistry and Physics, Plekhanov Russian University of Economics, Stremyannyi pereulok, dom 36, 117997 Moscow, Russia

**Keywords:** metal silsesquioxane, X-ray analysis, topological analysis, oxidation, alkanes, alcohols, *meta*-chloroperoxybenzoic acid (MCPBA)

## Abstract

New hexanuclear nickel(II) silsesquioxane [(PhSiO_1.5_)_12_(NiO)_6_(NaCl)] (**1**) was synthesized as its dioxane-benzonitrile-water complex (PhSiO_1,5_)_12_(NiO)_6_(NaCl)(C_4_H_8_O_2_)_13_(PhCN)_2_(H_2_O)_2_ and studied by X-ray and topological analysis. The compound exhibits cylinder-like type of molecular architecture and represents very rare case of polyhedral complexation of metallasilsesquioxane with benzonitrile. Complex **1** exhibited catalytic activity in activation of such small molecules as light alkanes and alcohols. Namely, oxidation of alcohols with *tert*-butylhydroperoxide and alkanes with *meta*-chloroperoxybenzoic acid. The oxidation of methylcyclohexane gave rise to the isomeric ketones and unusual distribution of alcohol isomers.

## 1. Introduction

Cage-like metallasilsesquioxanes (CLMSs) [1,2,3,4,5,6,7,8,9,10,11,12,13], being a family of polyhedra with inorganic (metal silicate-like) cores and organic environments, have been thoroughly investigated in the line of their potential application in catalysis [14,15,16,17]. Nevertheless, their capacities as catalysts of oxidation processes remain in the shadow until recent past. Then, some of us reported the first examples of the oxidation reactions catalyzed by copper(II) silsesquioxanes, possessing different types of cage geometry, namely cooling tower [18,19], globule [19,20], sandwich [20], and cylinder [21].

To use the advantage of good solubility of cage metallasilsesquioxanes in organic solvents and the ability of bringing unusual effects in the catalytic act due to specific structures of catalytic centers [19] we decided to study some other (not copper-containing) types of CLMSs. Here we present preliminary results on first examination of new Ni(II)-CLMS under oxidation conditions.

## 2. Results and Discussion

### 2.1. Synthesis

**Scheme 1:**
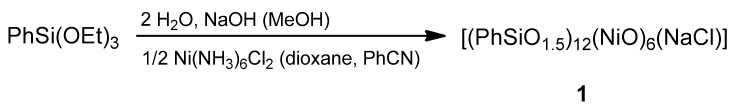
Synthesis of compound **1**.

Recently, some of us reported on ability of 1,4-dioxane molecules to serve as bridging linkers, combining individual CLMSs into an entire supramolecular system [20], and we were interested in the synthesis of new dioxane-CLMS complexes. Performing the synthesis of Ni-phenylsilsesquioxane [starting from PhSi(OEt)_3_] in dioxane-containing media allowed us to isolate (in 20% yield) a new cage-like hexanuclear product [(PhSiO_1.5_)_12_(NiO)_6_(NaCl)] (**1**) in the form of its adduct with dioxane/benzonitrile/water solvating ligands (PhSiO_1,5_)_12_(NiО)_6_(NaCl)(C_4_H_8_O_2_)_13_(PhCN)_2_(H_2_O)_2_ (Scheme 1).

### 2.2. Structure

**Figure 1:**
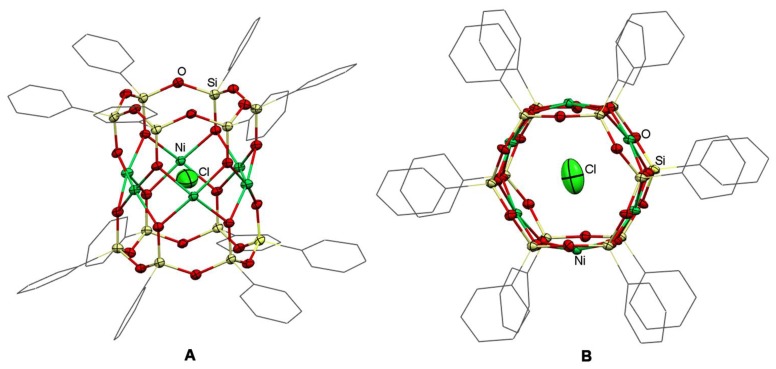
Molecular structure of compound **1** (**A**—side view, **B**—top view). Solvating ligands and counter ion Na^+^ are omitted for clarity.

The age skeleton of the product belongs to a CLMS of the cylinder type [9], characterized by the presence of three layers: two 12-membered siloxane cycles mutually connected through a metal-oxide (Ni_6_O_6_) cycle (Figure 1; see Supplementary Materials for more details).

**Figure 2:**
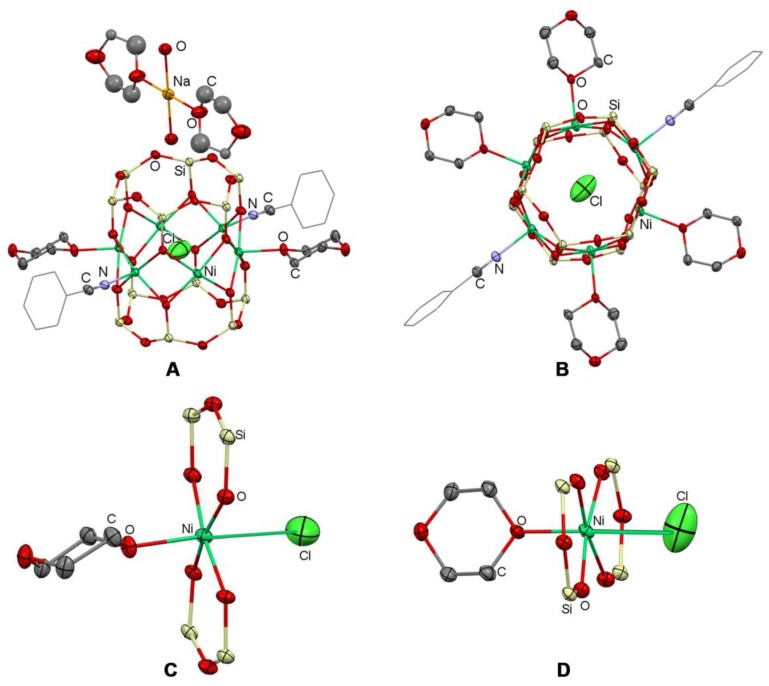
Structures **A** and **B**: compound **1** with solvating ligands (side and top views, respectively). Ph groups at silicon atoms are omitted for clarity; structures **C** and **D** is the illustration of distorted octahedral coordination of nickel ion. The anion Cl^‒^ and the oxygen atom of the coordinated dioxane molecule occupy an axial position, while the oxygen atoms of siloxanolate cycles form the equatorial plane of the coordinating octahedron.

The inner void of the cylinder moiety contains a chloride anion which occupies the crystallographic center of inversion. Each Ni(II) ion of **1** adopts distorted octahedral coordination. The axial positions of the octahedron are occupied by a Cl^‒^ anion and oxygen or nitrogen atoms of coordinated dioxane and benzonitrile molecules, respectively. Four nickel ions of **1** are coordinated by dioxane molecules, while the remaining two ions are bonded to benzonitrile molecules (Figure 2). Possibly, the donor ability of solvent molecules can govern the strength of Ni···Cl coordination bond and distortion of cylindric shape as consequence. Indeed, Ni···Cl distances are noticeably different [2.7231(7), 2.8269(8) and 2.9474 Å]. It is noteworthy, that shortest Ni···Cl distances correspond to nickel atoms coordinated by dioxane molecules.

This is just the second evidence that benzonitrile ligands could participate in aggregation of a CLMS structure. The first observation of such unusual coordination was presented by some of us [21] for the copper-containing CLMS.

### 2.3. Topological Analysis and Supramolecular Assembly

Following the procedure of a metal cluster notation [22] implemented into the ToposPro package (the Samara Center for Theoretical Materials Science, Samara, Russia) [23] we obtained that nickel atoms in compound **1** form in terms of the *N*D*k-m* notation the discrete *5*M*6-1*clusters, where 5 is the coordination number of topologically non-equivalent nodes, M denotes a discrete cluster, 6 is the number of metal atoms in the cluster, and 1 is a classification number to distinguish topologically-distinct clusters with equal NDk parameters. A database of topological representations of polynuclear nickel compounds [24] contains representatives of the nickel clusters with the *5*M*6-1* topology, and μ_6_-coordinated Hal^−^ and S^2−^ anions; recently, some of us have synthesized a nickel-silsesquioxane encapsulating the O^2−^ anion [13]. Nevertheless, to our knowledge, complex **1** is only the sixth known representative of Ni_6_ clusters with the *5*M*6-1* topology.

**Figure 3:**
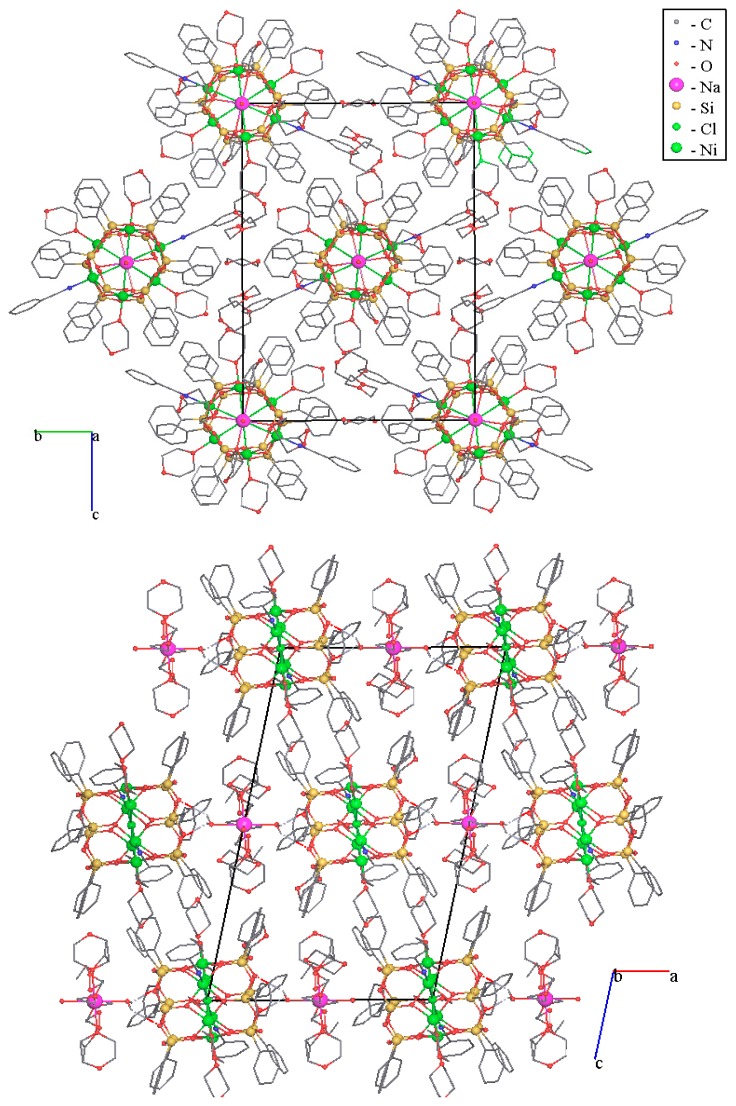
Crystal packing of **1**.

An additional attractive feature of synthesized complex is a formation of supramolecular structure where cage components are assembled into infinite chains (Figure 3) via H-bonds between water molecules bonded to sodium anions and oxygen atoms of siloxane cycles. The r(O···O) and ∠OHO are equal to 3.364(6)‒3.488(6) Å and 135.7°‒149.0°. The connection between ions is additionally supported by C-H···O interactions between 1,4-dioxane and silsesquioxane as short as 3.62(2) and 3.91(2) Å for r(O···C). As a consequence, cylinder cage fragments and complex cations Na(H_2_O)_2_(O_2_C_4_H_8_)_2_ share the same pseudo two-fold axis parallel to the [100]-crystallographic direction. The chains are packed as the hexagonal rod packing, with the distances between two-fold axes of 16.1 and 16.3 Å and non-parallel disposition of Ni_6_ metal rings. Only weak C‒H···O and C‒H···π bonding between neighboring chains, or chains and solvent molecules, were found. Worth noting is that the shortest distance between two oxygen atoms of 1,4-dioxanes connected with Ni is equal to 8.6 Å, which is only slightly longer than the distance between nitrogen atoms of 4,4′-bipyridine, at 7.1 Å. In principle, this means that bipyridine and its analogues can be used to obtain coordination polymers connected by linkers through d-metals even for bulky phenylsilsesquioxanes. This opportunity will be a subject of our further investigations.

### 2.4. Oxidations Catalyzed by Compound ***1***

**Figure 4:**
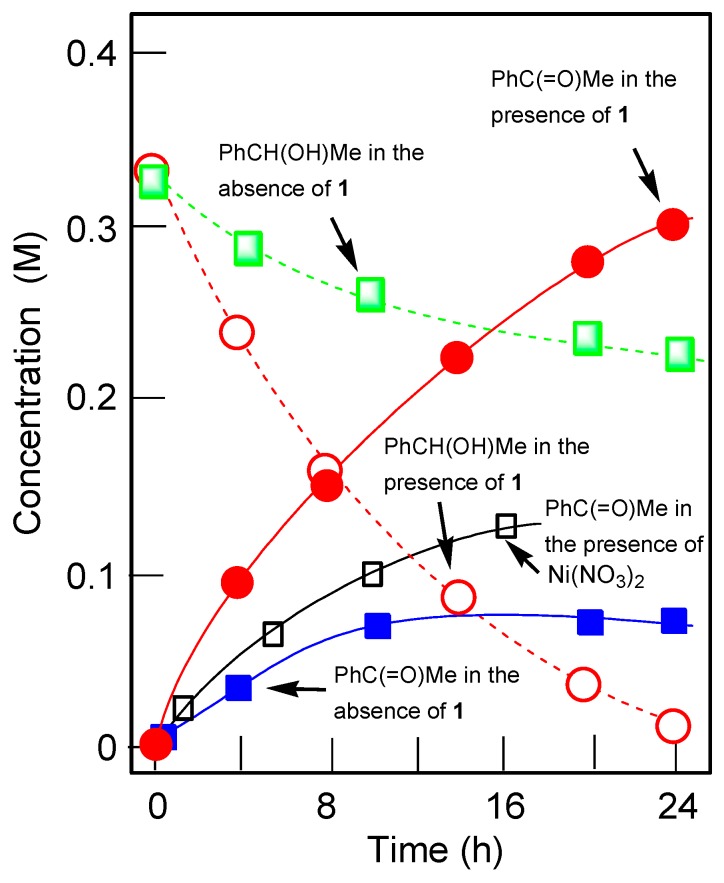
Formation of acetophenone with time in the 1-phenylethanol (0.33 M) oxidation with *tert*-butyl hydroperoxide (TBHP; 70%, aqueous, 1.65 M) in the absence and in the presence of compound **1** (5 × 10^−4^ M), as well as in the presence of Ni(NO_3_)_2_. Solvent was acetonitrile (total volume of the reaction solution was 5 mL); temperature was 70 °C.

Nickel complexes are known to catalyze certain oxidation reactions of hydrocarbons [25,26,27,28,29,30,31,32,33,34,35,36,37] and alcohols [38,39,40,41,42,43,44] by peroxides. We have tested the catalytic effect of compound **1** in oxidations with various oxidants. It turned out that **1** does not catalyze the oxidation of 1-phenylethanol or alkanes with hydrogen peroxide in acetonitrile solution. In contrast to H_2_O_2_, *tert*-butyl hydroperoxide oxidizes 1-phenylethanol at 70 °C to afford acetophenone in 90% yield (initial reaction rate *W*_0_ = 7 × 10^−6^ M s^−1^; initial TOF = 50 h^−1^) after 24 h. The kinetic curves of acetophenone accumulation shown in Figure 4 indicate the pronounced catalytic effect of compound **1**.

As can be expected, alkanes are less reactive in comparison with alcohols and only *meta*-choroperoxybenzoic acid (*m*-CPBA) turned out to be a good oxidant. Certain complexes of transition metals have been previously reported to oxidize alkanes with *m*-CPBA [27,28,29,32,33,34,36,45,46,47]. Complex **1** exhibited activity in the oxidation of cyclohexane with *m*-CPBA (Table 1). It can be seen that at 20 °C the reaction deceased after 15 min. The ketone/alcohol ratio is not changed in the chromatograms made before and after reduction of samples with triphenylphosphine. This indicates that cyclohexyl hydroperoxide is not formed in the course of the oxidation (for this simple method, see [48,49,50,51]). The yield of oxygenates was 24% and TON = 64, TOF = 256 h^−1^.

**Figure 5:**
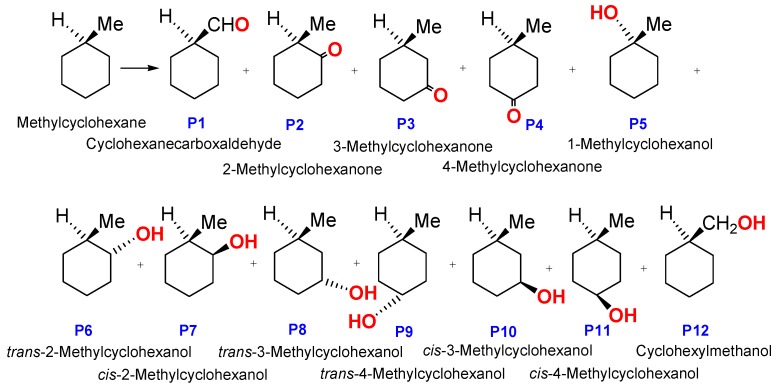
Products formed in the methylcyclohexane oxidation.

**Figure 6:**
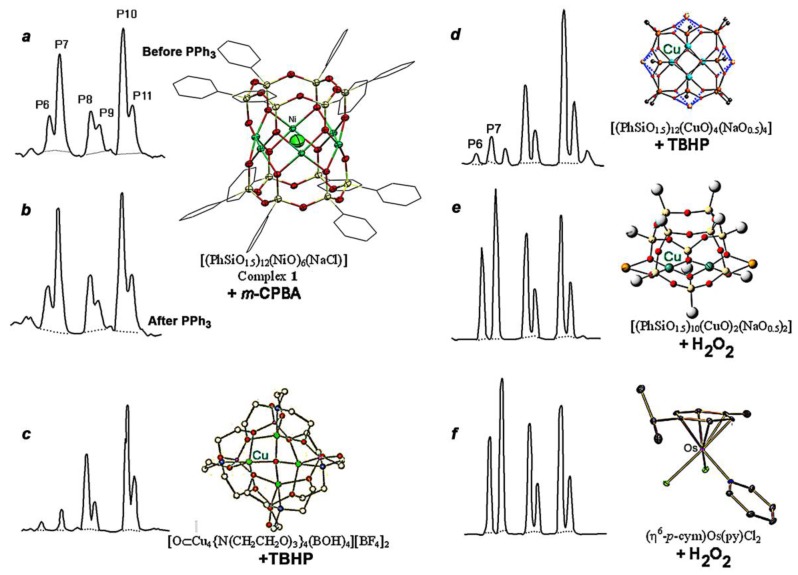
Chromatograms (fragments) of products formed in the methylcyclohexane oxidation by various systems: complex **1**/*m*-CPBA (**a**, **b**, this work); [O⊂Cu_4_{N(CH_2_CH_2_O)_3_}_4_(BOH)_4_][BF_4_]_2_/TBHP (**c**, [52]); [(PhSiO_1.5_)_12_(CuO)_4_(NaO_0.5_)_4_]/TBHP (**d**, [19]); [(PhSiO_1.5_)_10_(CuO)_2_(NaO_0.5_)_2_]/H_2_O_2_ (**e**, [19]); (η^6^-*p*-cym)Os(py)Cl_2_/py/H_2_O_2_ (**f**, [53]).

The oxidation of *n*-octane (0.12 M) with *m*-CPBA (0.13 M) in the presence of compound **1** (5 × 10^−4^ M) and co-catalyst HNO_3_ (0.05 M) at 60 °C during 3 h gave rise to the formation of a mixture of 2-, 3-, and 4-octanones (0.009, 0.009, and 0.008 M, respectively; yield 22%; TON = 52, TOF = 208 h^−1^). The oxidation of methylcyclohexane under similar conditions gave predominantly isomeric ketones (products **P2**–**P4**; M) and *tert*-alcohol **P5** (M; Figure 5 and Figure 6). Concentrations (M) of the isomers were the following: **P2** (0.0034), **P3** (0.0036), **P4** (0.0013), **P5** (0.012), **P6** (0.0003), **P7** (0.0008), **P8** (0.0004), **P9** (0.0002), **P10** (0.0009), and **P11** (0.0004); yield was 9% (TON = 47). It can be clearly seen that the ratio of isomeric alcohols in the case of this catalytic system (a, b) is different from that obtained earlier for other catalysts (c–f). Indeed, concentrations of isomers **P8** and **P9** is noticeably low in comparison with amounts of both **P6**, **P7** and **P10**, **P11**. This effect has not been found for other catalysts (c–f) and is apparently due to sterical hindrance around catalytic centers in **1**. Like in the cyclohexane oxidation, chromatograms of oxygenates obtained from methylcyclohexane (Figure 6a,b) before and after reduction with PPh_3_ are very similar and this indicates that alkyl hydroperoxides are also not formed in this experiment. 

The oxidation of *cis*-1,2-dimethylcyclohexane with *m*-CPBA in MeCN catalyzed complex **1** proceeds non-stereoselectively: the ratio of formed tertiary *trans* and *cis* alcohols *t*/*c* was 0.88 before reduction with PPh_3_ and 0.93 after the reduction (yield was 12% based on *cis*-1,2-DMCH; TON = 34). In the blank experiment (without complex **1**) the *t*/*c* ratio was 0.77 after reduction with PPh_3_ (yield was 5%).

## 3. Materials and Methods

### 3.1. Synthesis of Compound ***1***

Compound PhSi(OEt)_3_ and solvents were purchased from Acros Organics (Moscow, Russia) and were used as received.

Compound PhSi(OEt)_3_ (3 g, 12.48 mmol), water (0.45 g, 24.96 mmol) and NaOH (0.5 g, 12.50 mmol) in 20 mL of methanol were placed into a flask, equipped with a magnetic stirrer and condenser. After total dissolution of NaOH, the solution was heated to reflux for 1.5 h. Afterwards solution was cooled down to room temperature and mixed with 85 mL of dioxane. Then Ni(NH_3_)_6_Cl_2_ (1.4 g, 6.04 mmol) was added at once. Mixture was brought to reflux along with simultaneous distillation of the solution to remove methanol from reaction mixture. When 18 mL of distillate was collected, mixture was heated to reflux for additional 4 h and then left stirring at room temperature overnight. Then reaction mixture was filtered into an evaporation flask containing benzonitrile (8 mL). The flask was equipped with a septum and needle to allow solvents to evaporate under a slow current of nitrogen. Immediately after yellow-colored crystals began to form, the flask was transferred to the fridge and stored there until the crystal fraction growth (two weeks) ceased, as visually determined. A few selected single crystals were used for the X-ray study (for details, see below). Yield: 0.41 g, 20%; elemental analysis calcd (%) for [(PhSiO_1.5_)_12_(NiO)_6_(NaCl)]: Ni 17.12, Si 16.39, C 42.04, H 2.94, N 0; found (in a vacuum-dried sample): Ni 17.01, Si 16.30, C 42.64, H 3.07, N traces.

### 3.2. X-ray Diffraction Study

X-ray diffraction studies were carried out on Bruker APEX DUO diffractometer (Madison, WI, USA). The structure was solved by direct method and refined in anisotropic approximation against F2. The positions of hydrogen atoms were calculated from geometrical point of view and refined in isotropic approximation (the C‒H and O‒H distances and displacement parameters of hydrogen atoms are constrained). All calculations were carried out with SHELX (Gottingen, Germany) [54,55] and OLEX2 software (Durham, UK) [56]. The experimental parameters and crystal data are summarized in Table 2. 

### 3.3. Catalytic Oxidation of Alkanes and 1-Phenylethanol

Typically, the catalyst and the co-catalyst (nitric or trifluoroacetic acid) were introduced into the reaction mixture in the form of stock solutions in acetonitrile. The reactions of alcohols and hydrocarbons were carried out in air in thermostated Pyrex cylindrical vessels with vigorous stirring and using MeCN as solvent. The substrate (alcohol or hydrocarbon) was then added and the reaction started when the oxidant was introduced in one portion (CAUTION: the combination of air or molecular oxygen and peroxides with organic compounds at elevated temperatures may be explosive!). The reactions with 1-phenylethanol were analyzed by ^1^H-NMR method (solutions in acetone-*d*_6_; “Bruker AMX-400” instrument, 400 MHz, Billerica, MA, USA). Areas of methyl group signals were measured to quantify oxygenates formed in oxidations of 1-phenylethanol. As stated previously, the samples obtained in the alkane oxidation were typically analyzed twice (before and after their treatment with PPh_3_) by GC. This method (an excess of solid triphenylphosphine is added to the samples 10–15 min before the GC analysis) was proposed by one of us earlier [48,49,50,51]. Samples of the reaction mixture were analyzed by GC (Agilent 6890, Santa Clara, California, United States, N_2_ was carrier gas, FID) and GC-MS (Shimadzu QP-2010 Plus, Nishinokyo-Kuwabara-cho, Nakagyo-ku, Kyoto 604-8511, Japan; He was carrier gas); in both instruments the column was BP-20 (SGE; polyethyleneglycol −30 m × 250 μm × 0.25 μm). Assignment of peaks was made by comparison with chromatograms of authentic samples and by GC-MS.

## 4. Conclusions

Synthesized in this work complex (PhSiO_1,5_)_12_(NiO)_6_(NaCl)(C_4_H_8_O_2_)_13_(PhCN)_2_(H_2_O)_2_ represents very rare case of polyhedral complexation of metallasilsesquioxane with benzonitrile. The complex exhibited catalytic activity in oxidation of alcohols with *tert*-butylhydroperoxide and alkanes with *meta*-chloroperoxybenzoic acid. 

## Figures and Tables

**Table 1:** Oxidation of cyclohexane with *m*-CPBA catalyzed by complex **1** ^1^.

Entry	Time (min)	Reduction with PPh_3_	Cyclohexanone (M)	Cyclohexanol (M)
at 20 °C				
1	120	no	0.007	0.007
2		yes	0.005	0.006
3	300	no	0.009	0.006
4		yes	0.008	0.007
at 50 °C				
5	7	no	0.008	0.02
6		yes	0.007	0.017
7	15	no	0.009	0.022
8 ^2^		yes	0.009	0.023
9 ^3^		yes	0.0002	0.0003
10 ^4^		no	0.0005	0.0027
11 ^4^		yes	0.00004	0.00007
12	30	no	0.009	0.022
13		yes	0.009	0.022
14 ^3^		yes	0.0007	0.0007
15 ^4^		no	0.0005	0.002
16	60	no	0.009	0.023
17		yes	0.009	0.023
18 ^3^		yes	0.001	0.001

^1^ Conditions. Concentrations [1]_0_ = 5 × 10^−4^ M, [cyclohexane]_0_ = 0.46 M, [*m*-CPBA]_0_ = 0.13 M. Solvent MeCN, total volume of the reaction solution was 5 mL; ^2^ For this entry, TON = 64, TOF = 256 h^−1^; ^3^ Salt Ni(NO_3_)_2_ was used instead of catalyst **1**; ^4^ An experiment in the absence of any Ni compound.

**Table 2:** Crystal data and experimental parameters of the product.

Brutto Formula	C_138_H_17__8_ClN_2_NaNi_6_O_52_Si_12_
Formula weight	3437.54
T, K	120
Space group	P2_1_/n
Z	2
a, Å	16.1899 (12)
b, Å	18.2778 (14)
c, Å	27.093 (2)
β, °	101.513 (2)
V, Å^3^	7855.9 (10)
d_calc_, g℘cm^‒3^	1.456
μ, cm^‒1^	9
F(000)	3600
2θ_max_, °	50
Reflections collected	100,379
Independent reflections	23,101
Reflections with I > 2σ(I)	11,161
Parameters	942
R1 [for refl. with I > 2σ(I)]	0.0823
wR2	0.2050
GOF	1.006
Residual electron density, e·Å^‒3^(ρ_min_/ρ_max_ )	2.600/−2.575

## Supplementary Materials

Supplementary materials can be accessed at: http://www.mdpi.com/1420-3049/21/5/665/s1. CCDC 1471551 contains the supplementary crystallographic data for complex **1**. These data can be obtained free of charge from The Cambridge Crystallographic Data Centre via www.ccdc.cam.ac.uk/data_request/cif.
